# Cks1 Is Required for Tumor Cell Proliferation but Not Sufficient to Induce Hematopoietic Malignancies

**DOI:** 10.1371/journal.pone.0037433

**Published:** 2012-05-18

**Authors:** Susanne Kratzat, Viktoriya Nikolova, Cornelius Miething, Alexander Hoellein, Stephanie Schoeffmann, Oliver Gorka, Elke Pietschmann, Anna-Lena Illert, Jürgen Ruland, Christian Peschel, Jonas Nilsson, Justus Duyster, Ulrich Keller

**Affiliations:** 1 III. Medical Department, Technische Universität München, Munich, Germany; 2 Institute of Clinical Chemistry and Biochemistry, Technische Universität München, Munich, Germany; 3 Sahlgrenska Cancer Center, University of Gothenburg, Gothenburg, Sweden; University of Nebraska – Lincoln, United States of America

## Abstract

The Cks1 component of the SCF^Skp2^ complex is necessary for p27^Kip1^ ubiquitylation and degradation. Cks1 expression is elevated in various B cell malignancies including Burkitt lymphoma and multiple myeloma. We have previously shown that loss of Cks1 results in elevated p27^Kip1^ levels and delayed tumor development in a mouse model of Myc-induced B cell lymphoma. Surprisingly, loss of Skp2 in the same mouse model also resulted in elevated p27^Kip1^ levels but exhibited no impact on tumor onset. This raises the possibility that Cks1 could have other oncogenic activities than suppressing p27^Kip1^. To challenge this notion we have targeted overexpression of Cks1 to B cells using a conditional retroviral bone marrow transduction-transplantation system. Despite potent ectopic overexpression, Cks1 was unable to promote B cell hyperproliferation or B cell malignancies, indicating that Cks1 is not oncogenic when overexpressed in B cells. Since Skp2 overexpression can drive T-cell tumorigenesis or other cancers we also widened the quest for oncogenic activity of Cks1 by ubiquitously expressing Cks1 in hematopoetic progenitors. At variance with c-Myc overexpression, which caused acute myeloid leukemia, Cks1 overexpression did not induce myeloproliferation or leukemia. Therefore, despite being associated with a poor prognosis in various malignancies, sole Cks1 expression is insufficient to induce lymphoma or a myeloproliferative disease in vivo.

## Introduction

A central regulatory protein that exerts functions at the G1-S and G2-M transition of the cell cycle is the Cyclin-dependent kinase (Cdk) inhibitor p27^Kip1^ [1], [2]. Thus, complete loss of p27^Kip1^ leads to increased cell proliferation and overexpression of p27^Kip1^ arrests cells in G1 [3]–[6]. Because of the elemental role of p27^Kip1^ for ordered cell proliferation it also functions as a tumor suppressor and can be used as a prognostic factor in various malignancies [1], [7].

The regulation of p27^Kip1^ expression occurs mainly at the post-translational level. The current model involves phosphorylation of p27^Kip1^ at threonine 187 (T187) during G1 by Cyclin E/A-Cdk2 complexes, which marks p27^Kip1^ for recognition by the E3 ubiquitin ligase SCF^Skp2^ [1], [8]–[10]. SCF^Skp2^ requires the presence of the small protein Cyclin-dependent kinase subunit 1 (Cks1) that constitutes a part of the substrate binding surface, to allow efficient ubiquitylation of T187-phosphorylated p27^Kip1^ [11], [12]. Tissues from mice lacking *Cks1* accumulate p27^Kip1^ and exhibit proliferative defects [13]. Accordingly, *Cks1−/−* mice exhibit a small body size, resembling the size phenotype also seen in *Skp2−/−* mice [14]. *Cks1−/−* epithelial tissues however lack additional defects such as enlarged nuclei with polyploidy [14]. Cks1 is furthermore involved in Cdk activation and acts as a targeting protein for Cdks [15], [16], and as an essential regulator of mitosis. For example, Cks proteins are required for ubiquitylation and degradation of Cyclin A complexed with Cdc20 in pre-anaphase, which is required for mitotic progression [17].

The human *CKS1B* gene is located in a region of chromosome 1q that is frequently amplified in cancer. Cks1b has been found overexpressed in breast cancer [18], [19], as well as in a broad spectrum of other human malignancies [20]–[24]. In multiple myeloma (MM), an aggressive bone marrow cancer originating from terminally differentiated B cells, *CKS1B* is frequently overexpressed. Recent data showed that chromosome 1q21 amplification and elevated CKS1B protein expression were strongly correlated, and that nuclear CKS1B protein expression inversely correlates with p27^Kip1^ immunostaining. Importantly, CKS1B expression was associated with a shorter overall survival [25], [26].

We have previously shown that loss of *Cks1* and *Skp2* results in elevated levels of p27^Kip1^ in B cells, but that only loss of Cks1 resulted in a significant delay in lymphomagenesis of Eμ-*Myc* transgenic mice [27], [28]. We therefore hypothesized that Cks1 could have other oncogenic functions, possibly not related to its function in the SCF^Skp2^ complex. To address this we applied a Cks1 overexpression system in the B-cell compartment and in the pan-hematopoietic compartment of mice.

## Results

### Cks1 is overexpressed in various B cell malignancies

In Eμ-*Myc* mice, a model of the human Burkitt-Lymphoma [29], loss of Cks1 leads to a profound delay in lymphoma onset [27]. To investigate if lymphoid malignancies are associated with increased CKS1B transcript levels, we mined a publicly available gene expression array database (www.oncomine.org). We found the highest *CKS1B* transcript levels in multiple myeloma, and *CKS1B* was also highly expressed in Burkitt lymphoma, diffuse large B cell lymphoma, but not in chronic lymphocytic leukemia (CLL), a lymphoid disorder with a usually indolent clinical course and low proliferative activity, at least in the peripheral blood (Figure S1). Thus, various aggressive B cell malignancies are characterized by elevated *CKS1B* transcript levels.

**Figure 1:**
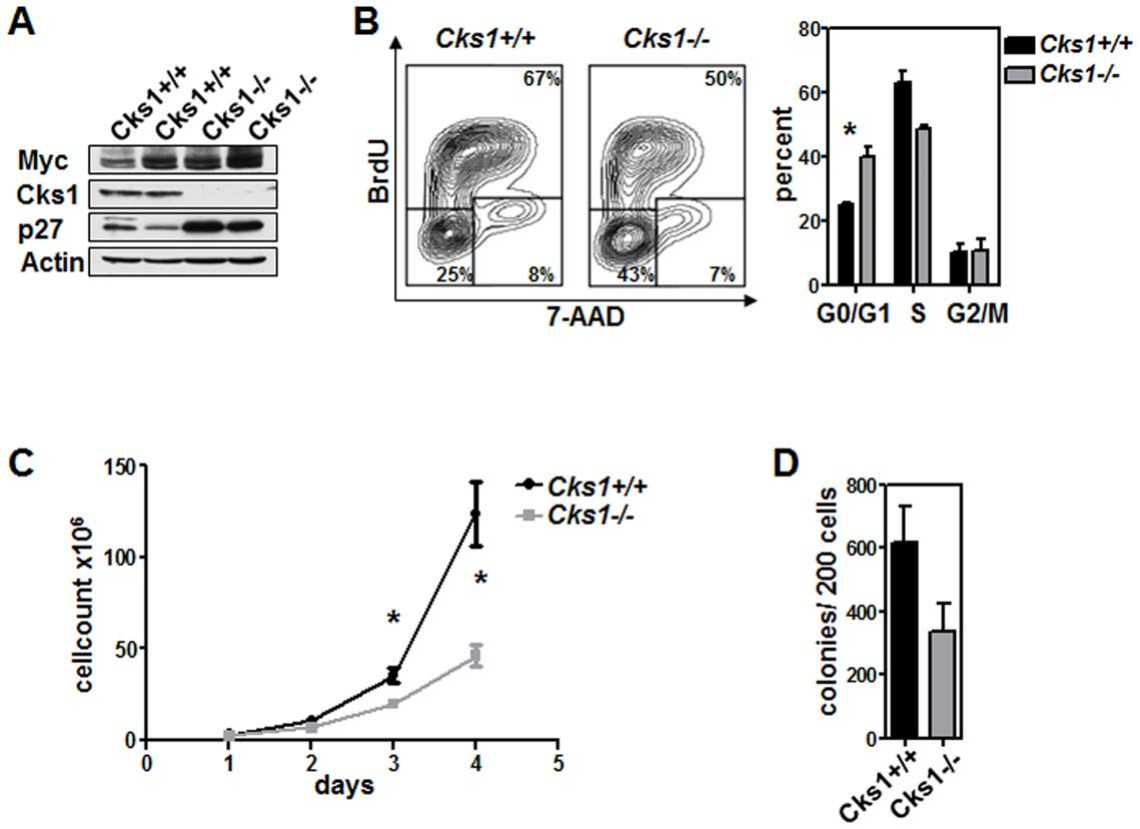
*Cks1*-deficient Eμ-*Myc* lymphomas display reduced proliferation and colony formation in vitro. Primary Myc-induced lymphoma cells of the indicated *Cks1* genotype were cultured *in vitro* until they were independent of stromal cell support. **A**, Immunoblot analysis of Myc, Cks1 and p27^Kip1^ protein levels, representative analysis. **B**, Left panel: Representative flow cytometry dot plot images of in vitro BrdU-labelled lymphoma cells of the indicated *Cks1* genotype. Right panel: the bars represent the mean ± standard error of the mean of the indicated cell cycle phase. * indicates p<0.05. **C**, Growth curve of *in vitro* cultured lymphoma cells of the indicated *Cks1* genotype. Cells were replated daily and the growth curve calculated based on the -fold increase in cell number starting with 1×10^6^ cells (day 1). The graph represents the mean ± standard deviation from 3 independent experiments. * indicates p<0.05. **D**, 200 Eμ-*Myc* lymphoma cells of the indicated *Cks1* genotype were plated in methylcellulose. The number of colonies was counted on day 7. The graph represents the mean ± standard deviation from 2 independent experiments.

Motivated by the clinical significance of Cks1 overexpression, we decided to continue our studies on the functions of Cks1 in B cell malignancies. One of the unanswered questions in our previous study [27] was if Cks1 loss only impacted cell proliferation in precancerous B cells or if the reduced proliferation was maintained in lymphomas that arose in Eμ-*Myc* mice. To address this we derived primary Myc-driven lymphoma cells of *Cks1+/+* or *Cks1−/−* genotype that were established in vitro. As expected *Cks1−/−* lymphomas showed highly elevated p27^Kip1^ protein levels with unaffected p27^Kip1^ transcript levels when cultured *in vitro* (Figure 1A and data not shown). Importantly, loss of Cks1 resulted in a significant growth reduction of the cultured lymphoma cells as assessed by BrdU incorporation and cell counting (Figure 1B, C). Furthermore, when grown in methylcellulose not supported by growths factors, we found an impaired formation of lymphoma colonies upon Cks1 loss (Figure 1D). Thus, even in established Myc-driven lymphomas grown *in vitro*, there is a marked impact on proliferation caused by Cks1 loss.

### Cks1 overexpression targeted to B cells is not sufficient to induce lymphoma

**Figure 2:**
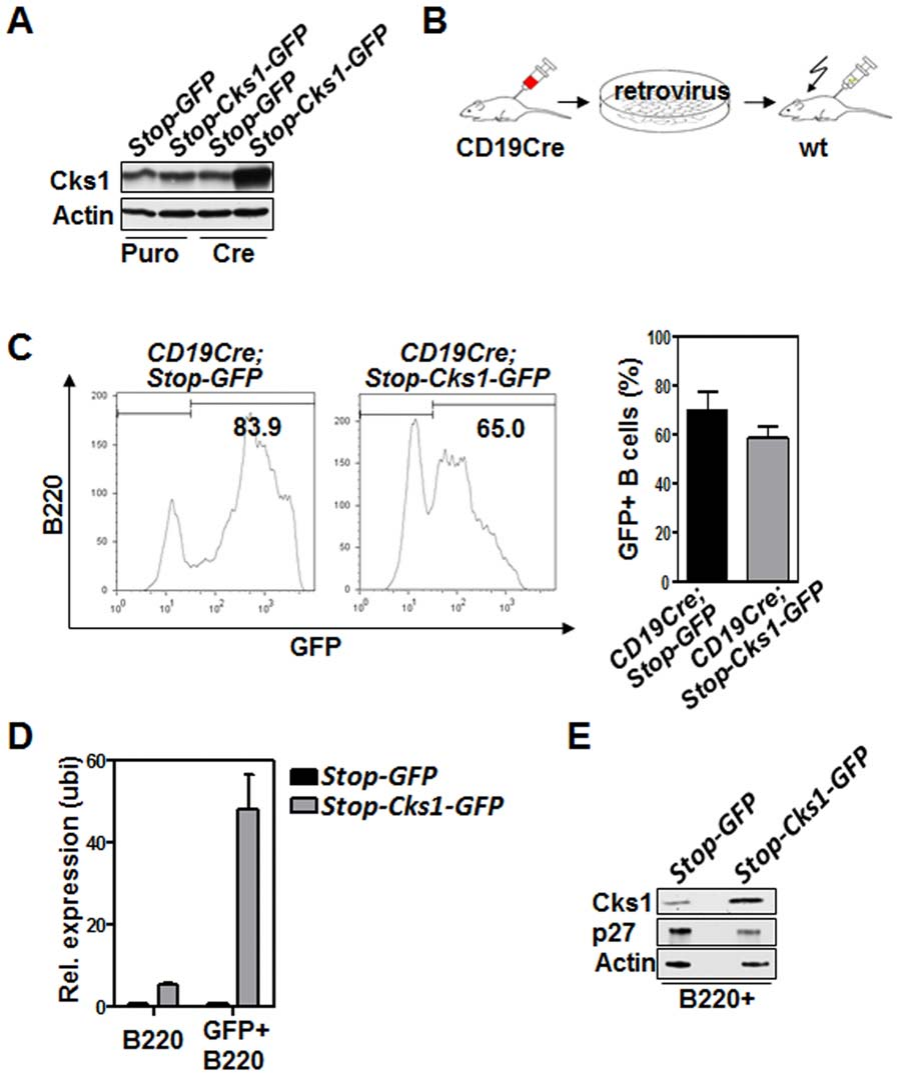
B cell-specific Cks1 overexpression results in elevated Cks1 protein levels. **A**, Immunoblot analysis of Cre-mediated conditional expression of Cks1 in retrovirally infected NIH-3T3 shows the tightness of the translational stop-cassette. Retroviral infection was performed 3 times in 12 hour intervals with 8 µg/ml polybrene. **B**, Depiction of the murine bone marrow retroviral transduction-transplantation model. 5FU-stimulated bone marrow cells from *CD19-Cre* transgenic mice were infected 4 times with retrovirus and then intravenously injected into lethally irradiated wild type (wt) recipient mice. **C**, Flow cytometric analysis of engraftment of GFP-positive B cells in the peripheral blood 28 days after bone marrow transplantation. Left panel: Histogram upon B220 staining. Right panel: Results of two different transplantations are combined. The bars represent the mean ± standard error (n = 7 mice for *Stop-GFP*, n = 9 for *Stop-Cks1-GFP*). One experiment was performed with 2×10^6^ transplanted cells and an infection rate of 15% (GFP positivity, n = 4 or 5 recipient mice), and one with 1×10^6^ cells and an infection rate of 45% (GFP positivity, n = 2 or 3 recipient mice). **D**, realtime PCR analysis of *Cks1* transcript levels in the indicated cells. *Stop-GFP* (n = 2), *Stop-Cks1-GFP* (n = 3). The bars represent the mean ± standard deviation. **E**, Immunoblot anaylysis of Cks1 protein levels in B220+GFP+ B cells. The B cells were FACS-sorted from n = 6 mice of each of the indicated treatment groups 50 days after transplantation.

**Figure 3:**
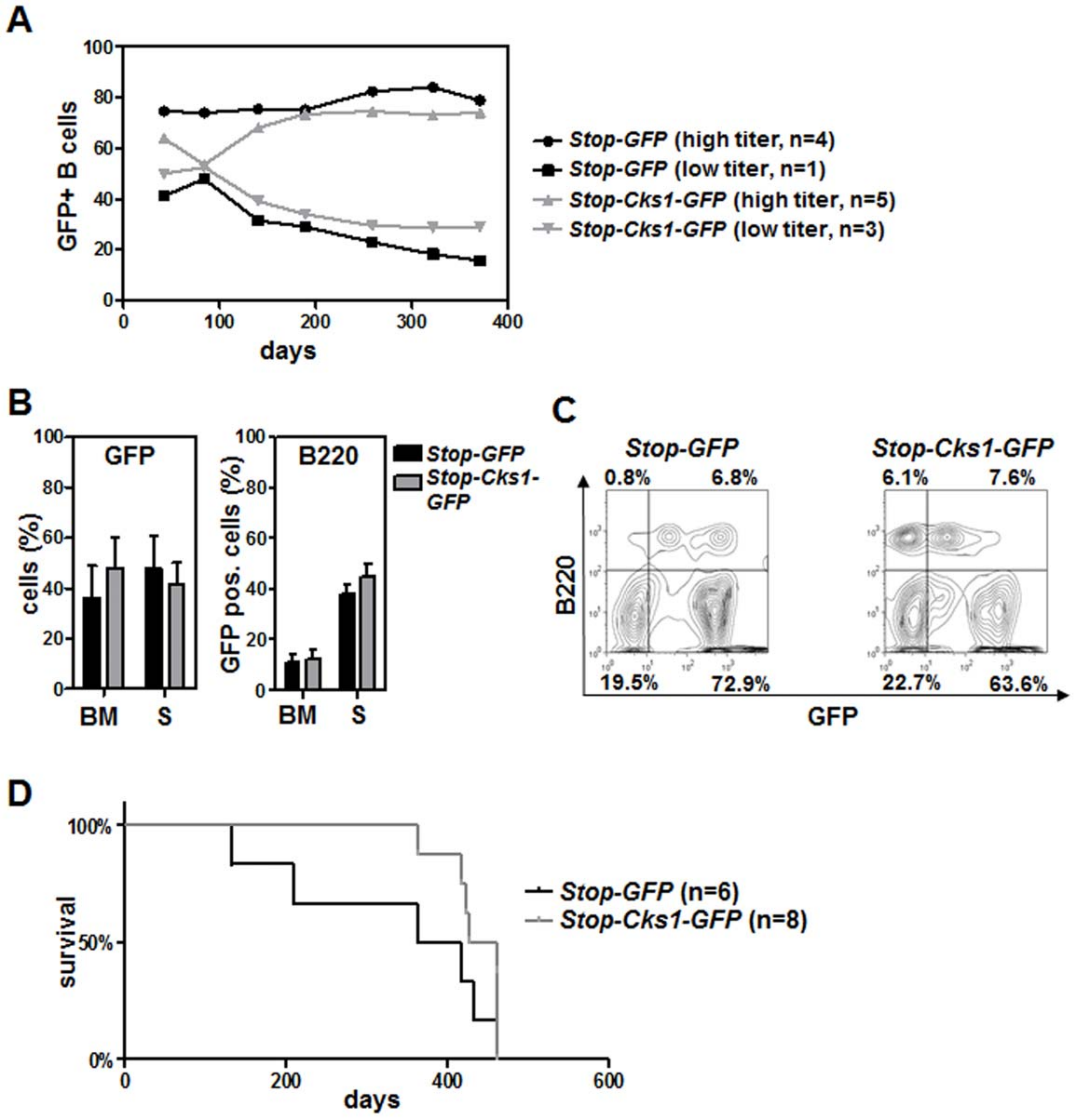
Targeted Cks1 overexpression is not sufficient to induce B cell lymphoproliferation or lymphoma. Data from two experiments (see Figure 2) are combined. One experiment with 2×10^6^ transplanted cells and an infection rate of 15% (n = 4 or 5 recipient mice) and one with 1×10^6^ cells and an infection rate of 45% (n = 2 or 3 recipient mice). **A**, Flow cytometric analysis of the percentage of GFP-positive B cells. Leucocytes were gated on B220+ cells. The analysis shows a divergence in the groups, some animals loose GFP positivity in the B cell compartment over time irrespective of the transduction efficiency. **B**, Left panel: Flow cytometric results of the bone marrow (BM) and spleen (S) for GFP positivity (of all cells, after lysis of erythrocytes). Right panel: percentage of GFP-positive cells in the B220 compartment of bone marrow (BM) and spleen (S). The analysis was performed on the day of indisposition and sacrifice. **C**, Representative images of flow cytometric analysis of GFP-positive B cells in the bone marrow. The genetic group is indicated. **D**, Survival of the *CD19-Cre;Stop-Cks1-GFP* group (median: 444 days) was identical to the survival of the *CD19-Cre;Stop-GFP* group (median: 391 days). All mice died from irradiation induced reasons but not B cell lymphoma.

The fact that tumor cells are unable to circumvent the dependence on Cks1 suggested that Cks1 could have broad effects on the cell cycle, or that the tumor cells are addicted to high levels of Cks1, akin to oncogene addiction. However, the likelihood that the general effects on cell cycle would be connected to p27^Kip1^ was hampered by the fact that Skp2 loss did not impact lymphomagenesis in Eμ-*Myc* mice, despite elevated p27^Kip1^ levels [28]. Therefore, to directly assess the putative oncogenic role of Cks1 in B cell lymphoma *in vivo* we cloned the *Cks1* coding sequence into a retroviral expression plasmid that allows Cre-mediated excision of a stop signal (Figure S2A, described in detail in Miething et al., submitted). To test whether Cre expression would indeed result in Cks1 overexpression we used NIH-3T3 cells expressing either MSCV-Cre-IRES-Puro (Cre) or Puromycin resistance alone (Puro) that were infected with MSCV-lox-stop-lox-Cks1-IRES-GFP or control virus. Elevated Cks1 protein levels were detected in Cre-expressing NIH-3T3 cells but not in control cells, indicating the functionality of the Cre-medited excision which leads to Cks1 protein expression (Figure 2A). Next, to test the transforming activity of Cks1 in vivo, we used a retroviral transduction-transplantation model [30]. Upon viral infection of *CD19-Cre* transgenic 5-FU mobilized donor bone marrow, the graft that was injected into the tail vein to reconstitute lethally irradiated recipients (Figure 2B). Two independent transplantations were established: one with low percentage (∼20%) of GFP infected graft cells and one with high percentage of GFP positivity in the graft (∼50%). The engraftment of both experiments was characterized by high GFP positivity in B cells after 28 days post transplantation (Fig. 2C). To ensure that the retroviral expression plasmid indeed resulted in a B cell-targeted expression of *Cks1* we verified elevated mRNA levels and protein levels in Cks-overexpressing B cells (Figure 2D, E). To analyze whether Cks1 expression in B cells would lead to a lymphoproliferation, blood samples were taken at regular intervals to examine changes in WBC counts and of GFP positivity in the transplanted mice. Both groups, the Cks1 group and the control group, showed heterogeneous patterns of GFP-positive B cells. Some animals had a very high amount of GFP positive cells during their life span and others showed reduced GFP positivity over time (Figure 3A). Importantly, WBC counts for both groups stayed in fairly normal range (data not shown). The analysis of mice with Cks1 overexpression targeted to B cells showed no difference in comparison to control mice with regards to either B cell frequency, a selective proliferation advantage or disadvantage of B cells expressing Cks1, or an altered B cell differentiation pattern (Figure S3). Importantly, there was no onset of either B cell proliferative disease or B cell lymphoma in the mice overexpressing Cks1 in the B cell compartment during the observation period. Eventually, the animals in both groups succumbed to irradiation-induced malignancies (Figure 3D; median survival: *Stop-Cks1-GFP* group, 410 days; *Stop-GFP* control group, 336.5 days). Post mortem analysis of the bone marrow, lymph nodes and spleen showed same amounts of GFP positivity and GFP positive B cells in the Cks1 group versus control (Figure 3B, C and data not shown). Thus, B cell-targeted overexpression of Cks1 could not induce a lymphoma or lymphoproliferative disease during the observation period.

### Cks1 regulates proliferation in oncogene-induced AML but is not sufficient to mediate myeloproliferation

**Figure 4:**
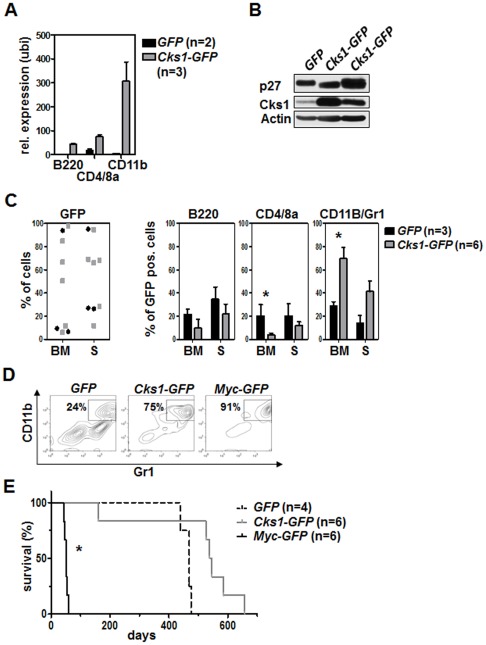
Ubiquitous Cks1 overexpression in the bone marrow is not sufficient to induce myeloproliferative disease or leukemia. 4×10^6^ GFP-positive cells were transplanted with an infection rate of 30%. **A**, Cks1 mRNA levels in the indicated bone marrow subsets of transplanted mice were analyzed by realtime PCR at day 160 after transplantation. Shown is the relative expression of Cks1 as compared to the B cell expression of the GFP mice as a control sample set at 1. *GFP* group: n = 2; *Cks1-GFP* group: n = 3. The bars represent the mean relative expression ± standard deviation. realtime PCR was performed in duplicates. **B**, Immunoblot analysis of the bone marrow of transplanted mice on the day of indisposition and sacrifice. **C**, The left panel shows the GFP positivity of the bone marrow (BM) and spleen (S). The three right panels show the percentages of B-, T- and myeloid cells. **D**, Representative flow cytometric dot blot images of the myeloid cell compartment of *GFP*, *Cks1-GFP* and *Myc-GFP* animals on the day of their sacrifice. **E**, Survival analysis of lethally irradiated recipient mice that were transplanted with *GFP*, *Cks1-GFP* or *Myc-GFP* infected bone marrow. None of the *GFP* or *Cks1-GFP* mice died of leukemia, while all *Myc-GFP* mice succumbed to AML. Median survival: *GFP* group, 469 days; *Cks1-GFP* group, 541.5 days; *Myc-GFP* group, 52 days. Only the survival of *Myc-GFP* group is significantly different from the other groups.

High Cks1 levels are a characteristic of various cancers and are also associated with low levels of the tumor suppressor p27^Kip1^ [25], [31]. To test whether Cks1 would induce malignancy when ubiquitously overexpressed in the bone marrow of lethally irradiated recipient mice we again performed bone marrow transplantations in a transduction-transplantation model, this time with a constitutive retroviral expression plasmid (Figure S2B). We achieved highly elevated ectopic Cks1 transcript and protein levels in the bone marrow of recipient mice (tested 160 days post transplantation, Figure 4A and after indisposition and sacrifice 4B). This resulted in an expansion of the myeloid compartment in bone marrow and spleen (Figure 4C) but we did not detect tumor induction upon ectopic Cks1 overexpression. Rather, both groups of mice succumbed to irradiation-induced secondary malignancies not associated with hematopoietic disease (Figure 4D, E, and data not shown).

**Figure 5:**
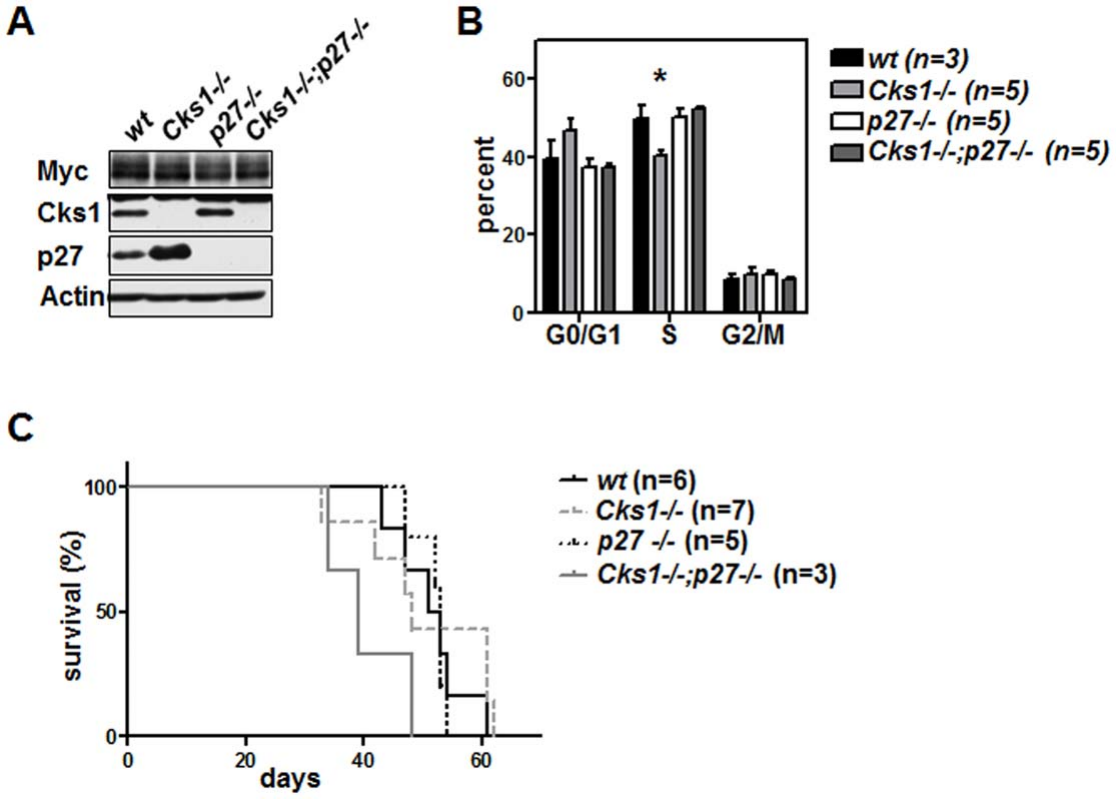
Cks1 regulates proliferation in oncogene-induced AML by suppressing p27^Kip1^. **A**, 5-FU-mobilized bone morrow from mice of the indicated genotype was infected with *Myc-GFP* virus and 2×10^6^ GFP-positive cells were transplanted with an infection rate of about 40% into lethally irradiated recipients. Immunoblot analysis of the indicated proteins was performed 3 weeks after transplantation. **B**, Flow cytometric anti-BrdU staining of GFP-positive Gr1/CD11b-positive bone marrow cells. 1 mg BrdU/g body weight was intraperitoneally injected 12 hours before bone marrow harvest. The bars represent the mean ± standard deviation of n = 3 or 5 individual mice per genotype. **C**, Survival curve of mice transplanted with *Myc-GFP*-infected bone marrow of the indicated *Cks1* and *p27^Kip1^* genotype. All mice died from AML. The median survival is not significantly different between the genotypes.

At variance with Cks1 overexpression, c-Myc overexpression using the same system resulted in acute myeloid leukemia (Figure 4D, E). To reveal if loss of Cks1 would impact this disease, we overexpressed c-Myc in hematopoietic progenitors from wild type and *Cks1* knockout mice (Figure 5A). To rule out effects of p27^Kip1^ we also transduced bone marrow cells from *p27^Kip1^* knockout and *Cks1;p27^Kip1^* double knockout mice with the c-Myc retrovirus. As expected from the studies in the Eμ-*Myc* mice [27] loss of *Cks1* resulted in a significant reduction of the percentage of premalignant Gr1/CD11b positive myeloid cells in S phase (Figure 5B), confirming that Cks1 is required to allow the full proliferative advantage conferred by the Myc oncoprotein. However, in the absence of p27^Kip1^ this proliferative deficiency was completely reverted as shown in *Cks1;p27^Kip1^* double knockout bone marrow-transplanted mice bearing ectopic Myc expression (Figure 5B). Surprisingly though, the effects on cell proliferation of either loss of Cks1 or its reversal by concomitant loss of p27^Kip1^ had no significant impact on the disease onset in the highly aggressive Myc-AML model (Figure 5C).

## Discussion

Cks1 is an essential component of the SCF^Skp2^ complex that controls among others the cell cycle inhibitor p27^Kip1^ [32], [33]. The function of Cks1 and Skp2 in lymphoma development is however not clearly understood as the correlative data from several studies indicates that sole accumulation of p27^Kip1^ is not the mechanistic explanation for the observed effects. First, only *Cks1* loss, but not *Skp2* loss, significantly delays lymphomagenesis, despite that both knockout mice have elevated p27^Kip1^ levels in the B cell compartment [27], [28], and despite the observation that *p27^Kip1^* loss dramatically accelerates Myc-induced lymphomagenesis [34]. *Skp2* loss does however impact tumorigenesis in a *Pten*-deletion model [35]. Second, overexpression of one of the SCF^Skp2^ complex components (Cks1 or Skp2) may not necessarily result in elevated activity of the whole complex and suppression of p27^Kip1^ levels. (see Figure 4B, and [36]). Still, Skp2 overexpression targeted to the prostate results in tumorigenesis [37]. Third, we can rescue a proliferation defect exerted by Cks1 loss in premalignant myeloid cells by removing p27^Kip1^, but changes in proliferation does not impact AML latency.

In this study we addressed the question whether or not Cks1 is an oncogene in the hematopoietic system. Although we cannot demonstrate that Cks1 itself is oncogenic, we found that Cks1 constitutes an important circuit in oncogene-driven proliferation. In this scenario, Cks1's major effect is to suppress p27^Kip1^ levels [13] and therefore concurrent loss of p27^Kip1^ results in the full reconstitution of Myc-induced proliferation in myeloid cells. Moreover, our data also suggests that Cks1 is more important for B cell lymphomagenesis than for AML development. Although the effects of Cks1 loss on cell proliferation in premalignant myeloid cells was slightly weaker than in B cell lymphomas, our results suggest a disconnect between effects on cell cycle progression and effects on tumorigenesis. If so, it is plausible that Cks1 is involved in both these processes.

Elevated Cks1 levels have been found in various malignancies including aggressive lymphomas, and Cks1 transcription has been shown upon oncogene overexpression [27]. In an effort to link elevated Cks1 levels and oncogenesis recent work established that Cks1 overexpression and binding of Cks1 to Cdk2 results in overriding the intra-S-phase checkpoint triggered by activated oncogenes. Cks protein overexpression thus likely constitutes one mechanism whereby premalignant cells can circumvent this DNA damage response barrier, conferring a proliferative advantage under stress conditions, and therefore contributing to tumor development [38]. In conclusion, Cks1 may serve several functions in supporting tumor progression: first, to allow cells to escape the DNA damage response that is triggered by oncogene activation and conditions of replicative stress. Second, to suppress p27^Kip1^ levels which allows increased tumor proliferation, a finding that corresponds well with the negative prognostic impact of low tumor cell p27^Kip1^ levels [1], [34]. Furthermore, and probably highly important for the outcome of tumor patients, p27^Kip1^ has been shown to inhibit tumor cell invasion [39]. In line with this finding, the absence of Cks1 resulted in p27^Kip1^ accumulation and loss of lymphoma dissemination [27].

## Materials and Methods

### Mice and tumor surveillance

*Cks1*-null mice (mixed background) [13] were bred with Eμ-*Myc* transgenic mice (C57BL/6) [29]. F_1_ offspring were then bred to gain *Cks1+/+* and *Cks1−/−* Eμ-*Myc* transgenic littermates to obtain primary lymphomas. Animals were observed for signs of morbidity and tumor development. Tumors were harvested after sacrifice of mice and cells were frozen viable. *Cks1*-null mice and *p27+/−* mice [4] were bred to obtain *Cks1−/−;p27−/−* mice. Recipient C57/Bl6 mice for transplantation experiments were purchased from Charles River Laboratories (Sulzfeld, Germany) or Harlan Laboratories (Eystrup, Germany) and were monitored for disease by serial peripheral blood analysis and daily physical examination. The animal experiments performed have been approved by the responsible regional authorities (#55.2-1-54-2531-48-08, Regierung von Oberbayern).

### Cell culture

Eμ-*Myc* lymphoma cells were established from single cell suspensions of tumors arising in Eμ-*Myc* mice. Lymphomas were cocultured on stromal cells (cell line EL08-1D2) [40] in RPMI (Gibco life technologies, Darmstadt, Germany) supplemented with 10% FCS (PAA Laboratories, Pasching, Austria), 1% Pen/Strep, 1% L-Glutamin, 0.1% Mercaptoethanol (all from gibco life technologies) and 10 ng/ml IL-7 (mIL7, R&D Systems). When steady growth conditions were achieved lymphoma cells were deprived from EL08 cells and IL-7. Phoenix ecotopic helper-free retroviral producer cells (G. Nolan, Stanford, CA, USA) and NIH/3T3 cells (DSMZ, Braunschweig, Germany) were maintained in DMEM (Gibco life technologies) supplemented with 10% FCS. Colony formation analysis was done with MethoCult M3234 (Stem Cell Technologies) for 7 days. Cells were cultured at 37°C with 5% CO_2_.

### Plasmids

MSCV-c-Myc-IRES-GFP was a gift of C. Schmitt (Berlin, Germany). MSCV-Cks1-IRES-GFP was cloned by digesting the MSCV-IRES-GFP plasmid with EcoRI and BglII (Fermentas, St. Leon-Roth, Germany) following a PCR for Cks1 with primers containing overhangs for EcoRI and BglII restriction (Omniscript RT Kit, Quiagen, Hilden, Germany). Ligation was performed using the Quick T4 DNA Ligase (New England Biolabs, Ipswich, England). The Stop element in MSCV-Stop-GFP contains the translational stop-codon ‘TGA’ in each reading frame leading to a termination of translation. It is flanked by loxP sites allowing a Cre-mediated excision (described in detail in Mieting et al., submitted). MSCV-Stop-Cks1-IRES-GFP was cloned in two steps. First using PCR primers with EcoRI and BglII overhangs to excise the Stop construct out of the MSCV-Stop-GFP plasmid and ligation into MSCV-IRES-GFP. Secondly we performed a PCR for Cks1 with BglII and XhoI overhangs following ligation.

### Generation of retrovirus and bone marrow transplantation

Retrovirus was obtained through transient infection of Phoenix E cells with lipofectamine 2000 (Invitrogen, Carlsbad, CA, USA) according to the manufacturer's instructions. NIH/3T3 cells were infected three times in 12 h intervals with retrovirus, supplemented with 8 µg/ml Polybrene (Sigma-Aldrich, Milwaukee, WI, USA). Bone marrow infection and transplantation was performed as described earlier [30]. Lethally irradiated (900 rad) female C57/Bl6 mice were transplanted with 1×10^6^ to 4×10^6^ GFP+ cells with infection rates from 15% up to 50% measured as GFP positive cells. Transplanted cells and infection rates were kept at same rates in single experiments.

### Flow cytometry and cell sorting

Antibodies for flow cytometry were purchased from BD Pharmingen or eBioscience (San Diego, CA, USA). Cells were stained in PBS (PAA, Pasching, Austria) containing 0.5% BSA (Roth, Karlsruhe, Germany) and antibodies were used 1 µl/1×10^6^ cells. BrdU assays were performed using the BrdU Flow kit (BD Pharmingen, San Diego, CA) according to the manufacturer's instructions. Analysis was performed with the Cyan ADP Lx P8 (Coulter-Cytomation; Beckman-Coulter, Krefeld, Germany), flow cytometric cell sorting with the MoFlo (Cytomation, Beckman Coulter).

### RNA preparation and analysis, real-time PCR

For reverse transcriptase quantitative PCR (qRT-PCR), RNA was prepared from flow cytometry-sorted bone marrow cells using the Dynabeads mRNA Direct Micro kit (Dynal, Oslo, Norway). cDNA was prepared using the Omniscript RT kit (Quiagen, Hilden, Germany). qRT-PCR was performed using an Applied Biosystems 7900HT (Carlsbad, CA) and the Power Sybr Green kit (Applied biosystems, Warrington, UK). Data analyses were performed with the ΔCt method, where ubiquitin served as the internal control. Sequences for primers are available from the authors upon request.

### Immunoblotting

Cell lysis for immunoblotting was performed with lysis buffer containing 50 mM Hepes (pH 7.5), 150 mM NaCl, 1 mM EDTA, 2.5 mM EGTA, 0.1% Tween-20 (all from Sigma-Aldrich, Deisenhofen, Germany) and protease inhibitors (Roche, Mannheim, Germany) followed by sonification [41]. After SDS-PAGE, proteins were transferred to PVDF membranes (Millipore Corporation Billerica, MA, USA) and probed with antibody. Anti-Cks1 was purchased from Invitrogen (Frederick, MD, USA), anti c-Myc from Santa Cruz Biotechnology Inc. (Santa Cruz, CA), anti-p27^Kip1^ from BD Transduction Laboratories, and anti-ß-Actin (Actin) from Sigma-Aldrich (St. Louis, MO).

### Statistical analysis

Statistical analyses were performed using the statistical functions of Excel or GraphPad Prism (GraphPad Software, La Jolla, CA). The bars shown represent the mean ± standard deviation (SD) or standard error of the mean (SEM). All statistical analyses were *t*-tests. Only *p* values<0.05 were considered statistically significant.

## Supporting Information

Figure S1 **Elevated** ***CKS1B*** **transcript levels in various B lymphoid malignancies.** A public database (www.oncomine.com) was searched for studies that compare *CKS1B* transcript levels in control tissue and samples from patients with B cell malignancies [35]. Shown is the log2 median-centered relative intensity of expression for *CKS1B* [reporter: 37347_at].
(PDF)

Figure S2 **Cks1 expression plasmids used.** **A**, Schematic depiction of the lox-stop-lox plasmid used (*Stop-Cks1-GFP*). The control plasmid used is labeled *Stop-GFP* in all Figures. **B**, Schematic depiction of the expression plasmid used (*Cks1-GFP*) for ubiquitous expression. The control plasmid used is labelled *GFP*, the MSCV-*Myc*-*IRES-GFP* plasmid is labelled *Myc-GFP*.
(PDF)

Figure S3 **B cell-specific overexpression of Cks1 does not lead to a change in B cell frequency, cell cycle state, or differentiation.** Experiments were performed 28 days after bone marrow transplantation of 2×10^6^ GFP-positive cells and an infection efficiency of about 40%. Left panel: Flow cytometric detection of the percentage of Cks1 overexpressing B cells versus controls in the lymphoid compartment of the bone marrow using GFP as a marker. Middle panel: Flow cytometric anti-BrdU staining of GFP-positive B220+ B cells. 1 mg BrdU/g body weight was intraperitoneally injected 12 hours before bone marrow harvest. The bars represent the mean ± standard deviation of n = 7 or n = 6 individual mice per group. Right panel: Flow cytometric measurement of the differentiation pattern of Cks1 overexpressing B cells versus controls (GFP+, B220+ cells).
(PDF)
