# Supplementary figures and images for: Cks1 Is Required for Tumor Cell Proliferation but Not Sufficient to Induce Hematopoietic Malignancies
Source: PLoS One. 2012 May 18;7(5):e37433. doi: 10.1371/journal.pone.0037433 (PMC3356264; doi:10.1371/journal.pone.0037433)

**Figure S1**

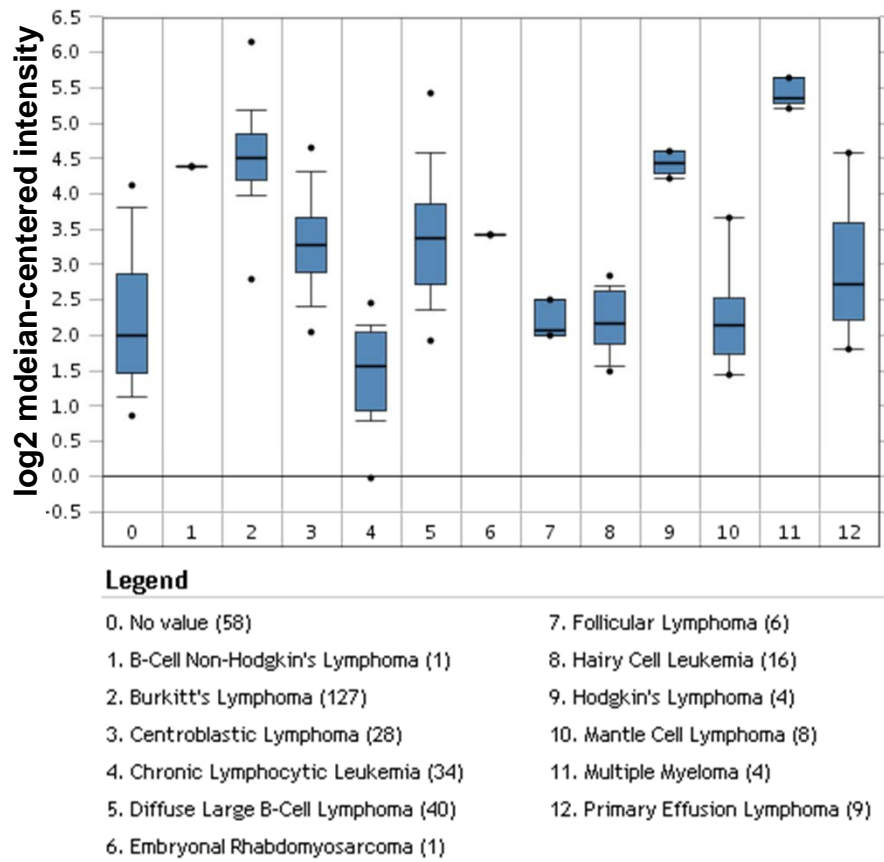

Supplement: Figure S1 — Elevated CKS1B transcript levels in various B lymphoid malignancies. A public database (www.oncomine.com) was searched for studies that compare CKS1B transcript levels in control tissue and samples from patients with B cell malignancies [35]. Shown is the log2 median-centered relative intensity of expression for CKS1B [reporter: 37347_at]. (PDF) [file pone.0037433.s001.pdf]

**Figure S2**

**A**

***Stop-Cks1-GFP***

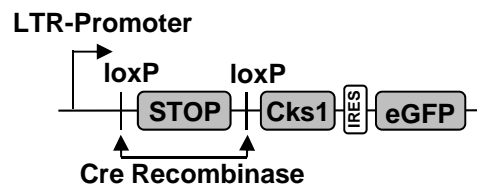

**B**

***Cks1-GFP***

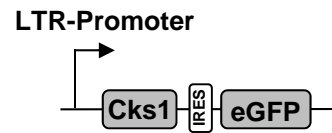

Supplement: Figure S2 — Cks1 expression plasmids used. A, Schematic depiction of the lox-stop-lox plasmid used (Stop-Cks1-GFP). The control plasmid used is labeled Stop-GFP in all Figures. B, Schematic depiction of the expression plasmid used (Cks1-GFP) for ubiquitous expression. The control plasmid used is labelled GFP, the MSCV-Myc-IRES-GFP plasmid is labelled Myc-GFP. (PDF) [file pone.0037433.s002.pdf]

**Figure S3**

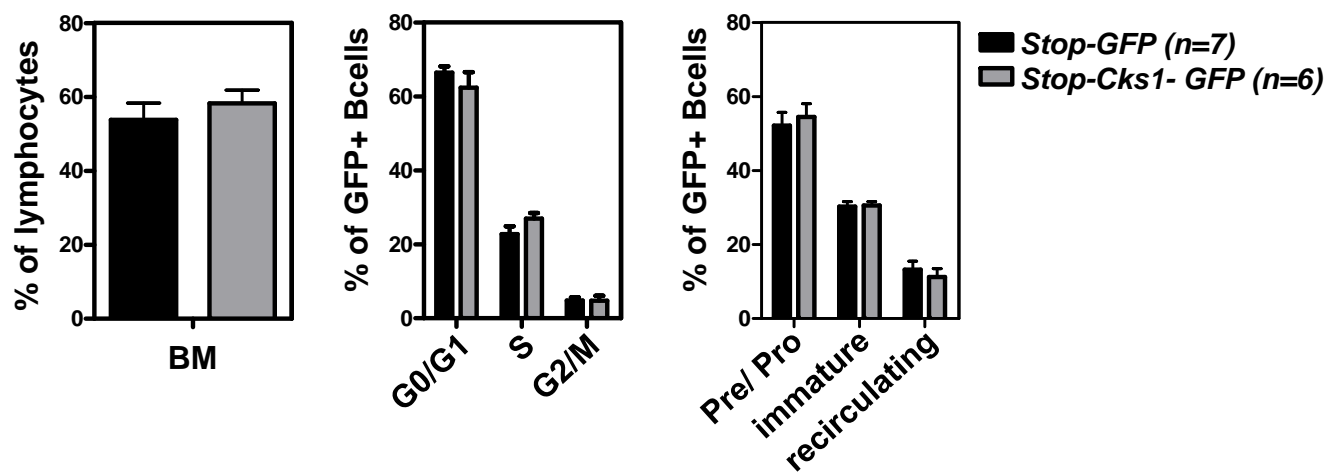

Supplement: Figure S3 — B cell-specific overexpression of Cks1 does not lead to a change in B cell frequency, cell cycle state, or differentiation. Experiments were performed 28 days after bone marrow transplantation of 2×10^6^ GFP-positive cells and an infection efficiency of about 40%. Left panel: Flow cytometric detection of the percentage of Cks1 overexpressing B cells versus controls in the lymphoid compartment of the bone marrow using GFP as a marker. Middle panel: Flow cytometric anti-BrdU staining of GFP-positive B220+ B cells. 1 mg BrdU/g body weight was intraperitoneally injected 12 hours before bone marrow harvest. The bars represent the mean ± standard deviation of n = 7 or n = 6 individual mice per group. Right panel: Flow cytometric measurement of the differentiation pattern of Cks1 overexpressing B cells versus controls (GFP+, B220+ cells). (PDF) [file pone.0037433.s003.pdf]
